# Extreme weight control behaviors among adolescent athletes: Links with weight-related maltreatment from parents and coaches and sport ethic norms

**DOI:** 10.1177/10126902211018672

**Published:** 2021-05-26

**Authors:** Véronique Boudreault, Marie-Pierre Gagnon-Girouard, Noémie Carbonneau, Sophie Labossière, Catherine Bégin, Sylvie Parent

**Affiliations:** 7321 Université de Sherbrooke, Canada; 14847 Université du Québec à Trois-Rivières, Canada; 14847 Université du Québec à Trois-Rivières, Canada; 248218 Université de Sherbrooke, Canada; 4440 Université Laval, Canada; Interdisciplinary Research Center on Intimate Relationship Problems and Sexual Abuse (CRIPCAS), Canada; NUTRISS Center, Institute of Nutraceuticals and Functional Foods, Canada; 4440 Université Laval, Canada; Interdisciplinary Research Center on Intimate Relationship Problems and Sexual Abuse (CRIPCAS), Canada

**Keywords:** disordered eating, sport, violence, parents, coaches, weight control, sport ethic, diet

## Abstract

The use of extreme weight-control behaviors is prevalent among adolescent athletes and may result from individual and sport-specific factors. Weight-related maltreatment from coaches and parents, and conformity to sport ethic norms have recently been linked to the use of extreme weight-control behaviors. This study aims to investigate the role of sport ethic norms and weight-related maltreatment from coaches and parents in the use of extreme weight-control behaviors among adolescent athletes. A sample of 999 French-Canadian athletes aged 14–17 years competing in a variety of sports completed an online survey assessing extreme weight-control behaviors, weight-related maltreatment from coaches and parents, and conformity to sport ethic norms. A total of 16.9% of the adolescent athletes reported having adopted extreme weight-control behaviors during their athletic careers. Extreme weight-control behaviors were significantly more prevalent among girls (19.75% vs 9.7% in boys) and weight-class-sport athletes (44%). In addition, 7.4% of the sample experienced at least one type of weight-related maltreatment by coaches or parents. Sex, weight-related neglect by coaches and parents, and weight-related psychological violence by coaches explained 24.4% of extreme weight-control behaviors variance. Indeed, participants who engaged in extreme weight-control behaviors experienced significantly more violence than the other participants did. In contrast, no differences were observed between people who engaged in extreme weight-control behaviors and those who did not due to conformity to sport ethic norms.

Although competitive sports participation during adolescence is related to many positive developmental outcomes, the value placed on performance also creates issues around safety and integrity. Among those issues, specific sports’ ideal body types (lean, thin, or muscular) are related to better performance outcomes in many sports settings (Muscat and Long, 2008), and may explain the use of extreme weight control behaviors (EWCB) by adolescent athletes. Because the value of performance is so inherent to competitive sports, such detrimental practices may be tolerated and even encouraged in sports environments (Coker-Cranney et al., 2020). In an organized sports setting, coaches and parents can act as the gatekeepers of the safety and integrity of adolescent athletes, or, on the contrary, be accomplices to the legitimization of EWCB because coaches and parents are also imbedded in this culture. In order to go beyond the minimization and legitimization of such practices in organized sports settings, more attention should be paid to the contribution of the norms surrounding each sport’s culture and the role of weight-related manifestations of maltreatment by coaches and parents in the adoption of EWCB.

## Extreme weight-control behaviors among adolescent athletes

Extreme weight-control behaviors (EWCB) such as severely restricted eating, fasting, and purging are prevalent among athletes (Joy et al., 2016; Sundgot-Borgen and Torstveit, 2010). These behaviors have detrimental health consequences and are associated with the development of eating disorders (ED) (Sundgot-Borgen et al., 2013). Although EWCB have mostly been studied among adult elite athletes, EWCB are especially alarming among adolescent athletes, who are in a critical physiological and psychological developmental period (Smink et al., 2012). In addition to the general societal pressure to be thin, the belief that a specific weight confers a performance advantage in many sports’ cultures encourages the use of EWCB in this population (Anderson and Petrie, 2012; Reel et al., 2013). Because adolescence is a formative period for the development of identity and self-esteem, adolescent athletes are at risk of adopting unhealthy behaviors to reach performance and body-image standards.

Only a few studies have investigated EWCB in adolescent athletes. Among European high-school-aged athletes, observed rates of EWCB range from 24% to 33% for female athletes and from 8% to 15% for male athletes (Martinsen and Sundgot-Borgen, 2013; Rosendahl et al., 2009). A review of 15 studies conducted among athletes aged 25 years and under suggests that EWCB is particularly prevalent in female athletes and athletes competing in weight-class sports (e.g. wrestling) compared with those competing in other sports (e.g. ball sports), including even aesthetic sports (Werner et al., 2013). In line with these results, a large study conducted with German adolescent elite athletes showed that youth competing in weight-dependent sports were more at risk of developing EWCB compared with those competing in other sports (Giel et al., 2016). Taken together, these results suggest that some subgroups of adolescent athletes (female athletes and weight-dependent-sports athletes) might be especially at risk of adopting EWCB. However, these conclusions are based on scant research conducted at elite levels, and thus do not capture the reality of adolescent athletes competing at development levels in organized sports settings.

So far, studies have mainly focused on individual vulnerabilities and personal-level risk factors for using EWCB (gender, elite-level participation, and body dissatisfaction), contributing to the vision that EWCB are symptoms of an individual pathology. Yet, the focus on individual risk factors for the use of EWCB limits the accountability of socio-cultural influences in the understanding and management of this problem (Papathomas, 2018). To this end, the possibility that some sport-specific factors can lead adolescent athletes to adopt EWCB needs to be seriously addressed.

## EWCB and its relationship to sport ethic

There is evidence that some unhealthy and abusive practices (EWCB, overtraining, use of performance-enhancing substances, and maltreatment) are legitimized in sports as a result of the dominant sports performance culture (Coker-Cranney et al., 2018; Parent et al., 2019; Waldron, 2015). According to Hughes and Coakley (1991), competitive sports culture praises athletes’ conformity to specific norms (sport ethic norms), including striving for distinction, accepting risks, playing through pain and injury, and accepting no limits in the pursuit of excellence. These authors argue that athletes who “overconform” to this sport ethic accept its norms uncritically and may engage in unhealthy behaviors to prove their dedication to their respective sports. Further studies show how overconformity can lead to dangerous practices such as playing while injured (e.g. Madrigal et al., 2015), substance use (e.g. Veliz et al., 2015), and disordered eating (e.g. Coker-Cranney et al., 2018).

Support for the association between EWCB and dedication and commitment from athletes toward their chosen sport comes from qualitative studies (Coker-Cranney and Reel, 2015; Coker-Cranney et al., 2018; Cosh et al., 2019; McGannon and McMahon, 2019; McMahon and Barker-Ruchti, 2017; Papathomas, 2018). For instance, interviewed collegiate wrestlers expressed that being able to control their weight was a source of pride and a means of demonstrating their striving for distinction (Coker-Cranney et al., 2018). The impact of the “slim-to-win” culture on the use of EWCB was also demonstrated through the testimonials of elite swimmers, who highlighted the role of the informal pressure from their sports culture in exacerbating their struggles with disordered eating (McGannon and McMahon, 2019; McMahon and Barker-Ruchti, 2017). Through interviews conducted among 73 male athletes from different sports disciplines, Atkinson (2011) observed how controlling weight became socially acceptable and even preferred by male athletes as they advanced within their sports culture. Although they are based on experiences collected from only a few athletes competing in very specific high-performance environments, these results clearly support the value of further investigating the influence of sport ethic norms on the use of EWCB in organized sports settings.

Recently, Parent et al. (2020) developed the conformity to the sport ethic scale (CSES), a questionnaire used to measure the conformity of adolescent athletes to three sport norms: self-sacrifice (e.g. I can do anything to prove my commitment), striving for distinction (e.g. I always try to be better than the other athletes), and refusing to accept limits (e.g. I refuse to accept my own limits when it comes to reaching my performance goal). This scale has been validated and developed with a large sample of adolescent athletes competing in organized sports settings. It is a promising tool for fostering a better understanding of the role of sport ethic norms in EWCB among adolescent athletes and for eventually adding quantitative evidence to that obtained from qualitative studies conducted with elite athletes.

## EWCB and its relationship to weight-related maltreatment

Because organized sports settings are deemed to be safe environments supervised by caring adults, one could expect that sport ethic norms and their association with legitimization of unhealthy behaviors and maltreatment would be discouraged by coaches, parents, and stakeholders. However, there is a growing body of evidence surrounding violent and neglecting behaviors perpetrated by coaches, parents, and other sporting agents (e.g. medical staff) on young athletes (Mountjoy et al., 2016; Parent et al., 2019). Results even suggest that athletes perceive maltreatment as “normal and as a necessary part of the game in sport, a way to achieve success, a way to protect themselves, or a result of the cult of performance and high expectations” (Fortier et al., 2020: 157). Maltreatment perpetrated by a person in authority includes sexual, physical, and psychological violence, as well as neglect or deprivation/lack of care (e.g. noticing a problem without intervening; Parent and Fortier, 2018). In relation to EWCB, the actions of forcing or asking a young athlete to adopt a self-destructive eating behavior in order to achieve an ideal weight for the sport is a manifestation of psychological violence, while allowing the use of EWCB without intervening is a manifestation of physical neglect (Fortier et al., 2020). Thus far, these “weight-related maltreatment behaviors” have mostly been studied as “perceived weight-related pressure” in the EWCB literature. Addressing these unacceptable practices as manifestations of maltreatment is warranted in order to overcome normalization of these behaviors in sports environments.

Pressure from coaches to lose weight was reported by athletes and associated with the use of EWCB (e.g. Anderson and Petrie, 2012; Arthur-Cameselle and Quatromoni, 2011; Martinsen et al., 2010). For example, in a study assessing dieting behaviors among adolescent athletes, 33% of boys and 13% of girls reported dieting when instructed to do so by their coach (Martinsen et al., 2010). Among 248 female college athletes, Coker-Cranney and Reel (2015) observed that athletes who reported more weight-related coach pressure (e.g. coach encouraging athletes to maintain a below-average weight, ascribing importance to body weight and appearance, or encouraging athletes to reduce weight) exhibited more disordered eating than athletes who reported less weight-related coach pressure. Athletes competing in aesthetic sports reported more weight-related coach pressure than did athletes in endurance and ball sports. This suggests that coaches may endorse and more actively contribute to the sports culture of “thin to win,” which emphasizes weight and shape. The authors concluded that athletes who overconform to sport ethic norms could uncritically accept weight-related pressure from their coach and use EWCB in response to this pressure.

While evidence indicates that parents can encourage the development of disordered eating among their children through their comments and behaviors (e.g. Rodgers and Chabrol, 2009), weight-related maltreatment from parents in sports has been largely overlooked. Results from the rare studies that have investigated the influence of parents on athletes’ concerns with eating and weight show that parents’ comments about their children's weight or eating habits can reinforce the pressure to be thin that is already in place in some sociocultural sports contexts (e.g. Francisco et al., 2013). Parents, like coaches, bear the responsibility of young athletes’ welfare as they provide important instrumental and emotional support in their child's sporting experience but they are also immersed in the culture of sports performance. Consequently, some parents may—intentionally or not—perpetrate weight-related maltreatment on their children.

## Aims of the study

The present study aims to investigate the role of sport ethic norms and weight-related maltreatment from coaches and parents (e.g. encouragement to adopt EWCB) in relation to EWCB among adolescent athletes. The first objective is to identify the prevalence of adolescent athletes who: (a) report having used EWCB in their sports careers and (b) have been victims of weight-related coach and parental maltreatment (psychological violence and neglect), according to each sport's category and competition level. The second objective is to identify the socio-cultural factors (i.e. conformity to the sport ethic norms and weight-related maltreatment from coach and/or parents) associated with the use of EWCB.

## Methodology

### Participants and procedures

All procedures of this project were approved by the ethics committee of the institution where the study was conducted (approval number 2014-131 Phase III/26-10-2016). Data were collected through an online survey developed by Parent et al. (2019), aiming to document the general experience of adolescent athletes. Participants completed an anonymous survey hosted by a secure website (Qualtrics). The athletes signed a consent form electronically before starting the survey. The completion time ranged from 30 to 45 min.

Participants represent a convenient sample of 999 French-Canadian adolescent athletes (722 girls and 277 boys) aged between 14 and 17 years (mean = 15.68 years, SD = 1.16 years) competing in a variety of sports and at different levels in organized sports settings. Participants were recruited on a voluntary basis through different strategies such as mailing through their organization (regional league, club, or sport team), distribution of flyers in sport competitions and advertising in Facebook. The recruitment was part of a larger study assessing different types of maltreatment in the experiences of adolescent athletes in sport. Interested participants were invited either through a hyperlink or by typing an address to access the survey. Finally, athletes’ sport participation characteristics are presented in Table 1. The type of sport was based on the Sundgot-Borgen and Larsen (1993) classification: ball games (soccer, volleyball, basketball, ice hockey, American football, badminton, baseball, lacrosse, kinball, rugby, tennis, ultimate frisbee, handball, softball, ringuette, and table tennis), endurance sports (swimming, speed skating, rowing, track and field, canoe/kayak, cross-country skiing, biathlon, triathlon, and cycling), aesthetic sports (cheerleading, dance, synchronized swimming, gymnastics, and figure skating), technical sports (equestrian, fencing, sailing, golf, diving, alpine skiing, shooting, archery, trampoline, curling, and ski jumping), weight-dependent sports (boxing, weightlifting, powerlifting, karate, taekwondo, and wrestling), and other sports.

**Table 1.** Characteristics of sport participation in the total sample (*N* = 999).

Type of sport		Competition level		Weekly sports practice (h)	
Ball games	51.0 (509)	Local or regional	27.9	<5	24.0
Endurance sports	19.9 (199)	Provincial	50.0	6–10	36.7
Aesthetic sports	16.0 (160)	National	17.1	11–15	20.4
Technical sports	6.4 (64)	International	5.1	16–20	12.1
Weight-class sports	5.0 (50)			>20	6.7
Other sports	1.7 (17)				

*Note*: Percentage (frequency).

### Measures

In the survey, EWCB was measured using one item asking the participant to rate on a four-point Likert scale (0 = *never*; 1 = *rarely*, *1–2 times*; 2 = *sometimes, 3–10 times*; 3 = *often, more than 10 times*) the frequency with which they have voluntarily used an extreme method (fasting, excessive exercise, use of vomiting, laxatives, diet pills, diuretics) to reach the ideal weight for their sport. A dichotomous score was created by considering participants who have never adopted EWCB (0 = never) and those who had experienced it at least once (1 = rarely to often).

To assess participants’ experiences with weight-related psychological violence and neglect, items from the coach version and the parent version of the violence toward athletes questionnaire (Parent et al., 2019) were used. Coaches’ weight-related psychological violence and neglect were assessed through two questions (i.e. “Did your coach force or ask you to use one of these methods to reach the ideal weight in your sport?” and “Did your coach know you were using one of these methods to reach the ideal weight for your sport but did not intervene?”). Parents’ weight-related psychological violence was assessed through the same two questions, starting with “Did one of your parents….” Participants rated the frequency with which they experienced each type of maltreatment on a four-point Likert scale, where 0 = *never*; 1 = *rarely*, *1–2 times*; 2 = *sometimes, 3–10 times*; and 3 = *often, more than 10 times*.

The CSES (Parent et al., 2020) was used to assess the degree of conformity to the norms of the sport ethic. This scale, developed and validated in French on a sample of adolescent athletes, comprises 20 items and results in a three-factor structure: (a) self-sacrifice (e.g. I am willing to do anything to prove my commitment); (b) striving for distinction (e.g. I always try to be better than the other athletes); and (c) refusing to accept limits (e.g. I refuse to accept my own limits when it comes to reaching my performance goal). Participants rated their level of agreement with each statement on a four-point Likert scale ranging from 1 = strongly disagree to 4 = strongly agree. The internal consistency of these subscales is aligned with alpha coefficients ranging from 0.72 to 0.85. Correlations between these three subscales were statistically significant and varied between *r* = 0.40, *p *< 0.001, and *r* = 0.52, *p *< 0.001.

### Data analysis

Within the framework of this study, descriptive, comparative, and predictive analyses were carried out using version 25.0.0.2 of SPSS software. The confidence level of 95% was selected (*p* ≤ 0.05) in order to reject the null hypothesis. To describe the sample and meet the first objective, descriptive analyses (frequencies, mean, and standard deviation) were utilized. In addition, multinominal chi-square tests were calculated to compare the prevalence of EWCB and types of parental or coach maltreatment among sports category, competition level, and sex. For the second objective, a multiple binary logistic regression analysis was performed to identify the sociocultural factors (conformity to the sport ethic norms and weight-related maltreatment from coach and/or parents) that distinguish participants who reported having used EWCB from those who did not. To ensure that the postulates of the multiple binary logistic regression are respected, descriptive analyses and Pearson's correlations among all the variables of interest were performed to identify the presence of extreme cases or multicollinearity. For the multiple binary logistic regression, the variables that did not contribute significantly to the model (*p* > 0.05) were removed in order to present the most parsimonious model possible.

## Results

For the first objective, prevalence of EWCB and weight-related coach and parental maltreatment (psychological violence and neglect) among adolescent athletes were computed, according to their sports category, competition level, and gender. Results show that during their athletic careers, significantly more girls presented at least one EWCB than boys did (19.7% vs 9.7%) (*x*^2^(1, *n* = 999) = 14.02, *p* = 0.001, Cramer's *V* = 0.12). Moreover, prevalence of EWCB was statistically different depending on the sports categories (*x*^2^(5, *n* = 999) = 31.38, *p* = 0.001, Cramer's *V* = 0.18), with participants practicing a weight-class sport being more at risk of experiencing EWCB than others. However, the prevalence of participants who experienced EWCB was not statistically different according to competition level (*p *> 0.05).

As shown in Table 2, 16.9% of the sample reported the use of EWCB during their athletic careers; 4.1% of the participants experienced weight-related psychological violence by their coach; 1.3% experienced weight-related psychological violence by their parents; 3.9% experienced EWCB-related neglect by their coach; and 1.8% presented EWCB-related neglect by their parents. In addition, 7.4% of the sample (8.4% of girls and 4.7% of boys) experienced at least one of these types of maltreatment during their sports careers. Girls were significantly more likely than boys to report maltreatment by their parents or their coach (*x*^2^(1, *n* = 999) = 4.12, *p* = 0.042, Cramer's *V* = 0.06). In addition, 75.7% of adolescent athletes who had experienced at least one form of weight-related maltreatment have presented EWCB during their athletic careers. Thus, participants who experienced a form of weight-related maltreatment were significantly more likely to have presented EWCB than did participants who did not experience any weight-related maltreatment (*x*^2^(1, *n* = 999) = 196.32, *p* = 0.001, Cramer's *V* = 0.44). However, in the presence of coach/parent maltreatment, girls and boys did not differ significantly in terms of the proportion of EWCB (*x*^2^(1, *n* = 999) = 0.36, *p* = 0.55, Cramer's *V* = 0.07).

**Table 2.** Prevalence of extreme weight-control behaviors (EWCB) and weight-related maltreatment from parents and coaches.

	*N*	EWCB	Coach		Parent/step-parent	
			Psychological violence	Neglect	Psychological violence	Neglect
Sport category						
Technical	64	17.2 (11)	4.7 (3)	3.1 (2)	3.1 (2)	3.1 (2)
Endurance	199	17.1 (34)	2.5 (5)	4.0 (8)	0.5 (1)	0.5 (1)
Aesthetic	160	19.4 (31)	6.3 (10)	3.8 (6)	1.3 (2)	2.5 (4)
Weight class	50	44.0 (22)	22.0 (11)	18.0 (9)	8.0 (4)	10.0 (5)
Ball games	509	13.4 (68)	2.4 (12)	2.6 (13)	0.8 (4)	2.8 (14)
Other	17	17.6 (3)	0.0 (0)	5.9 (1)	0.0 (0)	5.9 (1)
Competition level						
Local/regional	352	17.3 (61)	2.0 (7)	2.3 (8)	0.3 (1)	2.0 (7)
Provincial	419	16.0 (67)	4.8 (20)	4.3 (18)	1.7 (7)	2.9 (12)
National/international	220	17.7 (39)	5.5 (12)	5.0 (13)	2.3 (5)	3.6 (8)
Sex						
Girls	722	19.7 (142)	4.7 (34)	4.3 (31)	1.4 (10)	3.0 (22)
Boys	277	9.7 (27)	2.5 (7)	2.9 (8)	1.1 (3)	1.8 (5)
*N* total	999	16.9 (169)	4.1 (41)	3.9 (39)	1.3 (13)	2.7 (27)

*Note*: Percentage (frequency).

The prevalence of participants who experienced at least one incident of weight-related maltreatment by parents or coaches does not statistically differ by sex or competition level (*p *> 0.05). In contrast, the prevalence of psychological violence (*x*^2^(5, *n* = 999) = 48.57, *p* = 0.001, Cramer's *V* = 0.22) or neglect (*x*^2^(5, *n* = 999) = 29.25, *p* = 0.001, Cramer's *V* = 0.17) by coaches and the prevalence of psychological violence (*x*^2^(5, *n* = 999) = 21.40, *p* = 0.001, Cramer's *V* = 0.15) or neglect (*x*^2^(5, *n* = 999) = 14.52, *p* = 0.013, Cramer's *V* = 0.12) by parents differ significantly among the sports categories. Participants in weight-class sports were more likely to have experienced a form of weight-related maltreatment by their parents or their coach.

For the second objective, socio-cultural factors (i.e. conformity to the sport ethic norms and weight-related maltreatment from coach and/or parents) associated with the use of EWCB were analyzed. The correlation matrix is presented in Table 3.

**Table 3.** Correlations between extreme weight-control behaviors (EWCB), sport ethic norms, and weight-related maltreatment by coaches or parents (*N* = 989).

Sport ethic		1	2	3	4	5	6	7	8	9
	1. Strive for distinction	1	0.45**	0.38**	0.06	0.07*	0.10**	0.02	0.05	0.22**
	2. Self-sacrifice	0.45**	1	0.51**	0.07*	0.10**	0.06	0.01	0.08**	−0.00
	3.Refusing to accept limits	0.38**	0.51**	1	0.05	0.08*	0.06	0.06	0.06	0.05
Weight-related maltreatment	4. Psychological violence—coach	0.06	0.07*	0.05	1	0.43**	0.29**	0.34**	0.32**	−0.05
	5. Neglect—coach	0.07*	0.10**	0.08*	0.43**	1	0.34**	0.44**	0.38**	−0.03
	6. Psychological violence—parent/step-parent	0.10**	0.06	0.06	0.29**	0.34**	1	0.53**	0.25**	−0.01
	7. Neglect—parent/step-parent	0.02	0.01	0.06	0.34**	0.44**	0.53**	1	0.30**	−0.03
	8. EWCB's presence/absence	0.05	0.08**	0.06	0.32**	0.38**	0.25**	0.30**	1	−0.12**
	9. Sex	0.22**	−0.00	0.05	−0.05	−0.03	−0.01	−0.03	−0.12**	1

*Note*: bilateral correlation * *p *≤ 0.05; ** *p *≤ 0.01.

The three sport ethic norms were moderately to strongly correlated with conformity (*r* = 0.38; 0.45; 0.51) (Cohen, 1988). Of those three variables, conformity to strive for distinction was weakly but positively correlated with neglect by coaches (*r* = 0.07, *p *≤ 0.05) and with weight-related psychological violence by parents (*r* = 0.10, *p *≤ 0.001). In addition, conformity to self-sacrifice was weakly but positively correlated with weight-related psychological violence perpetuated by coaches (*r* = 0.07, *p *≤ 0.05) and with weight-related neglect by coaches (*r* = 0.10, *p *≤ 0.001). Further, conformity to refusing to accept limits was weakly but positively correlated with weight-related neglect by coaches (*r* = 0.08, *p *≤ 0.05). As such, the more adolescent athletes presented high conformity to one of the sport ethic norms, the more likely they were to experiencing weight-related maltreatment from their coach or parents. Finally, of these three sport ethic norms, only conformity to self-sacrifice was significantly correlated with the presence of EWCB during the adolescent athlete's sports career (*r* = 0.08, *p *≤ 0.01).

Moreover, experiencing one form of weight-related maltreatment by coaches or parents was positively associated with the experiencing another form of weight-related maltreatment by coaches or parents. Indeed, weight-related psychological violence and neglect by coaches were positively and moderately correlated (*r* = 0.43, *p *≤ 0.01). Psychological weight-related violence perpetrated by parents was significantly but moderately correlated with weight-related psychological violence by coaches (*r* = 0.29, *p *≤ 0.01) and with weight-related neglect by coaches (*r* = 0.34, *p *≤ 0.01). Weight-related neglect by parents was moderately to strongly correlated with weight-related psychological violence by coaches (*r* = 0.34, *p *≤ 0.01), weight-related neglect by coaches (*r* = 0.44, *p *≤ 0.01), and weight-related psychological violence by parents (*r* = 0.53, *p *≤ 0.01). Also, the types of weight-related maltreatment perpetrated by parents or coach were all significantly and moderately correlated with the presence of EWCB (*r*_s_ ranging from 0.25 to 0.38, all *p*_s _≤ 0.01). Finally, sex is mildly to moderately correlated with the sport ethic norm “Strive for distinction” (*r* = 0.22, *p *≤ 0.01) and with EWCB (*r* = −0.12, *p *≤ 0.01). Overall, girls were less prone to striving for a high distinction and were more prone to presenting EWCB during their athletic careers than were boys.

To observe which variables better predict the presence of EWCB, a multiple binary logistic regression was performed. The three sport ethic norms were removed from the model because they did not contribute significantly (*p *> 0.05). The model fit the data well, with a fairly high classification rate of 86.7%. As shown in Table 4, the variables included in this model alone explain 24.4% of the variance of EWCB among participants.

**Table 4.** Presence of extreme weight-control behaviors (EWCB) according to the presence of weight-related maltreatment by coaches or parents.

	*N*	Sex	Psychological violence (coach)	Neglect (coach)	Neglect (parent/step-parent)	Constant	Nagelkerke Pseudo *R*-squared	Classification rate %
Model	999	0.44** (0.25)	8.04** (0.43)	18.35** (0.53)	11.95** (0.63)	0.39* (0.31)	0.244	86.7

*Note*: The entries correspond to standardized regression coefficients with their standard errors in parentheses. *p ≤ 0.01 **p ≤ 0.001.

Conformity to sport ethic norms did not significantly differentiate participants, who presented EWCB from those who did not. Participants whose coaches had been negligent were 18.35 times more at risk of having presented EWCB during their careers; whereas if the coach had perpetrated psychological violence, participants were 8.04 times more likely to have presented EWCB during their athletic careers. Further, participants with negligent parents/step-parents were 11.95 times more likely to have presented EWCB during their athletic careers than were participants with non-negligent parents. Finally, boys were 2.23 times less at risk than girls of adopting EWCB during their athletic careers.

## Discussion

The first objective of this study was to identify the prevalence of (a) adolescent athletes who report adopting EWCB in their sports careers and (b) who have been victims of weight-related coach and parental maltreatment (psychological violence and neglect), according to sports category and competition level. A total of 16.9% of adolescent athletes (19.7% of girls and 9.7% of boys) competing in an organized sports setting reported the use of EWCB at least once in their sports careers. These results are similar to the prevalence observed in other studies conducted among European elite adolescent athletes, ranging from 24% to 33% for female athletes and from 8% to 15% for male athletes (Martinsen and Sundgot-Borgen, 2013; Rosendahl et al., 2009). This once again suggest that female athletes engage in disordered eating more than male athletes, perhaps because they still face additional sociocultural and sport-specific pressure to change their weight and appearance (Reel et al., 2013). That 7.4% of participants (8.4% of girls and 4.7% of boys) reported having been a victim of weight-related psychological violence or neglect by coaches and parents during their athletic careers is a new finding: To the best of our knowledge, no previous study had investigated the frequency of these specific types of maltreatment. EWCB and weight-related maltreatment seem to affect athletes at all levels of competition. This suggests that, despite the gravity of such practices for the health, integrity, and safety of adolescent athletes, EWCB and weight-related maltreatment occur in organized sports settings, environments that are supposed to provide security and opportunities for positive youth development.

Regarding sports categories, nearly one out of every two (44%) of athletes competing in weight-class sports reported having adopted EWCB at least once in their sports careers, which is consistent with findings of the few studies addressing EWCB in young athletes (e.g. Giel et al., 2016; Werner et al., 2013). This study thus adds to the evidence of the adverse effects of dangerous weight-control practices in this subgroup of young athletes during a determining period in their development. The current study also brings new insight to this problem, highlighting the presence of weight-related psychological violence and neglect by coaches (40%) and parents (18%) in this type of sport. The weight-class sports environment thus appears to represent a risky setting for weight-related maltreatment of adolescent athletes. The absence of a significant proportion of athletes using EWCB in aesthetic and endurance sports is surprising, given previous studies showing the high rate of disordered eating in these sports disciplines known to emphasize weight and shape (e.g. Rosendahl et al., 2009; Smolak et al., 2000). It would be misleading, though, to conclude that controlling weight and reaching a desired body standard are not promoted in these organized sport settings. In fact, in the current study, extreme methods of controlling weight were assessed. These methods are related to more severe preoccupations with weight and the resulting development of ED. The observation of EWCB may have precluded the identification of participants who adopted more moderate risky behaviors, such as downsizing food portions or frequent weighing. Moreover, it is possible that EWCB and disordered eating happen later in athletes’ careers, as a result of prolonged exposure to a culture promoting specific weight and body-shape ideals (McGannon and McMahon, 2019; Papathomas and Lavallée, 2014).

The second objective of the present study was to identify the socio-cultural factors (i.e. conformity to the sport ethic norms and weight-related maltreatment from coach and/or parents) associated with the use of EWCB. A noteworthy finding is that almost three-quarters of the adolescent athletes (all sports combined and no significant gender difference) who had experienced weight-related maltreatment from their coaches and/or parents reported that they had engaged in EWCB during their athletic careers. Moreover, having experienced one form of weight-related maltreatment by coaches or parents was positively associated with having experienced another form of weight-related maltreatment by their coach or parents. This result adds to the literature about maltreatment in sports, showing that the physical and psychological integrity and safety of some adolescent athletes is threatened by weight-related maltreatment at home and in organized sports. More precisely, sex, weight-related neglect by parents and coaches, and psychological violence by coaches explains 24.4% of EWCB variance, indicating that weight-related maltreatment represents a risk factor for the use of EWCB. Of those variables, the two that best indicate the presence of EWCB are weight-related neglect by coaches followed by weight-related neglect by parents. These findings once again demonstrate the powerful influence of coaches’ attitudes and behaviors and highlight the severe consequences of maltreatment of young athletes (Arthur-Cameselle and Quatromoni, 2011; Vertommen et al., 2018). These results also speak to the importance of the coaching relationship in adolescence, which is known to grow in intensity compared with the parent–child relationship at this age (Fredricks and Eccles, 2004). The present study supports the idea that weight-related coach maltreatment and pressure are part of a complex socio-cultural problem caused by the legitimization of dangerous weight-control practices or even ED in sports settings (Arthur-Cameselle and Quatromoni, 2011; Coker-Cranney and Reel, 2015). It also means that the problem of maltreatment in sports needs to be more systematically addressed in relation to EWCB. It is encouraging, however, to observe that weight-related maltreatment from coaches and parents was not commonly reported by adolescent athletes in the current study (7.4% of participants), especially given that it is a significant determinant of the use of EWCB.

Interestingly, only parents’ weight-related neglect and not parents’ weight-related psychological violence were significantly associated with the use of EWCB. This non-significant result can be explained by the small reported prevalence of weight-related psychological violence from parents. However, the fact that some parents witness their children practicing EWCB and do not intervene means that they are complicit in these practices and they too may adhere to the dominant sports performance culture. In qualitative research conducted by Kerr and Stirling (2012), interviews conducted with parents illustrated how they become accustomed to the sports culture and become silent witnesses to abusive coaches’ practices. Parents studied also expressed guilt and discomfort (with some even crying during interviews) upon seeing their children being victims of emotional abuse by coaches without intervening, highlighting the powerful and subtle influence of the sports performance culture (Kerr and Stirling, 2012). Even if adolescents tended to withdraw from their parents as a need to build their autonomy, parents still play a determining role in their sports experience (Fredricks and Eccles, 2004) and act as important models for adolescents development of a healthy relationship with eating and body image (Hansson et al., 2017). That being said, more subtle and implicit forms of violence often receive less attention than explicit psychological and physical maltreatment but that does not mean that they are less damaging. Attention to more subtle forms of weight-related maltreatment, such as neglect, from authority figures in the context of organized sports practices is thus warranted if we want to protect adolescent athletes from socio-cultural influences related to the use of EWCB.

Contrary to what was expected, conformity to sport ethic norms was not significantly associated with the presence of EWCB among adolescent athletes; only self-sacrifice norms were significantly correlated with EWCB. Athletes who reported having engaged in EWCB were thus more likely to conform to the self-sacrifice norm. Striving for distinction and refusing to accept personal limits do not appear to be norms triggering the use of EWCB in this sample of adolescent athletes competing in organized sports settings. While it is too early to draw clear conclusions based on this one study, it can be hypothesized that, in opposition to “self-sacrifice,” “striving for distinction” and “refusing to accept [one's] own limits” are norms that may lead athletes to healthily push themselves to excellence. Indeed, athletes could more healthily strive for distinction and constantly push their own limits to reach their full potential. In support of this affirmation, a study about perfectionism in athletes showed that the dimension of striving for perfection (as opposed to the dimension of negative reactions to imperfection) was adaptative and associated with lower competitive anxiety and higher self-confidence during competitions (Stoeber et al., 2007). This hypothesis has yet to be confirmed, since other forms of “self-directed violent behaviors” such as doping and playing while injured might result from overconformity to these norms (Coker-Cranney et al., 2018). Moreover, athletes showing more evidence of over-control were found to be more at risk of presenting disordered eating in previous studies (e.g. Labossière and Thibault, 2020). To this end, it makes sense that EWCB require self-sacrifice, in that these extreme behaviors (e.g. restrictive eating, fasting, and purging) are forms of self-directed violence (Mountjoy et al., 2016; Parent and Fortier, 2018). Lastly, the fact that conformity to sport ethic norms was weakly associated with weight-related maltreatment is another, albeit weak, demonstration of the legitimization of violence and maltreatment in the dominant sports culture. It also adds support to the idea that athletes who overconform may be more vulnerable to being victims of maltreatment, perhaps because they are seen as more submissive and ready to prove their dedication to abusive coaches and parents (Hughes and Coakley, 1991; Parent and Fortier, 2018).

## Conclusion

The findings indicate that EWCB are especially prevalent in weight-related organized sports settings, and highlights the links among conformity with sport ethic norms, the presence of weight-related maltreatment by coaches or parents, and the presence of EWCB in adolescents. In the current study, conformity to sport ethic norms is not significantly associated with the use of EWCB in adolescent athletes, even if those norms were slightly significantly correlated with EWCB, which was probably due to the large sample used. Rather, it appears that the occurrence of weight-related maltreatment is most strongly linked to EWCB. Therefore, it seems that socio-cultural norms might more subtly influence an adolescent athlete's personal use of EWCB in organized sports setting through the external influence of weight-related maltreatment. This observation supports the value of moving from an individual pathology conceptualization of EWCB and disordered eating to a more comprehensive account of socio-cultural factors affecting these problems in sports. By specifically studying weight-related maltreatment (i.e. not perceived pressure), the present study affirms the recognition of coaches’ and parents’ abusive behaviors as specific forms of maltreatment in sports. It also brings important insight to the literature around EWCB, showing that this problem cannot be understood only from a unilateral vision of an individual pathology. Instead, it has to be seen as a problem resulting from complex interactions of factors, including sport-specific socio-cultural factors contributing to legitimizing and normalizing maltreatment and unhealthy practices even in young athletes.

The results also suggest that EWCB are multidetermined, meaning that other variables not considered in the present study may contribute to the variance of EWCB. For instance, peers and teammates are collectively an important source of social influence during adolescence, because adolescents tend to distance themselves from authoritarian figures (parents, teachers, and coaches) and become more affiliated with their peers (Chan et al., 2012). One study showed that pressure (e.g. modeling of eating disordered behaviors) from teammates had a significant influence on young athletes’ use of EWCB and development of ED (Arthur-Cameselle and Quatromoni, 2011; Arthur-Cameselle et al., 2017). In a review of violence and maltreatment in sports, other athletes were reported as the main offenders (as opposed to coaches and parents) (Parent and Fortier, 2018), and it was demonstrated that team norms were related to hazing (Waldron, 2015). Studying the influence of weight-related peer pressure on EWCB could yield a more comprehensive overview of the influence of weight-related maltreatment from significant others on EWCB.

This is the first study assessing EWCB among a sample of adolescent athletes aged between 14 and 17 years competing in organized sports at different levels (not just an elite level). This is also the first study examining EWCB in relation to weight-related maltreatment from coaches and parents and conformity to sport ethic norms, allowing better recognition of the contribution of socio-cultural factors to the problem of EWCB in sports. The large sample considered made it possible to increase the statistical power and limit type-1 errors by minimizing the number of analyses. However, every study has limitations and this one is no exception. Its transversal nature and the statistical analyses carried out preclude our establishing whether the socio-cultural characteristics identified precede or rather result from EWCB in adolescent athletes. Further, the absence of cut-off scores made it impossible to assess overconformity; only conformity to sport ethic norms was possible, which limited the conclusions drawn. It would have been interesting to observe whether presenting sport norms overconformity would increase the risk of adopting EWCB.

These findings must be replicated with other samples and, ideally, a longitudinal study would be important for observing the short- and long-term repercussions of EWCB experienced in adolescence (e.g. abandonment of sport, injuries, disordered eating, ED, or other mental health disorders in adulthood). Such studies should also include other offenders, such as peers and teammates. Lastly, given the interplay between the sports culture, maltreatment in sports, and EWCB, it would be interesting to study if other forms of maltreatment (i.e. not only weight-related) are related to the use of EWCB. To this end, the use of new validated questionnaires assessing conformity to the sport ethic (Parent et al., 2020) and maltreatment in sports (Parent et al., 2019) will allow future researchers to measure the presence of EWCB in relation to these sport-specific socio-cultural factors.
